# LncRNA TMPO-AS1 suppresses the maturation of miR-335-5p to participate in polycystic ovary syndrome

**DOI:** 10.1186/s13048-021-00848-3

**Published:** 2021-07-30

**Authors:** Fang Hou, Jie Li, Jie Peng, Zhenghua Teng, Jun Feng, Weiwei Xia

**Affiliations:** Department of Obstetrics and Gynecology, Suzhou Wuzhong People’s Hospital, No.61, Dongwu North Road, Suzhou City, Jiangsu Province 215128 P. R. China

**Keywords:** Polycystic ovary syndrome, TMPO-AS1, miR-335-5p, Maturation

## Abstract

**Background:**

TMPO-AS1 is a recently characterized oncogenic lncRNA in ovarian cancer. Its role in other ovary diseases is unknown. This study explored its role in polycystic ovary syndrome (PCOS).

**Methods:**

Follicular fluid was extracted from both PCOS patients and controls. The levels of TMPO-AS1 and mature and premature miR-335-5p were analyzed by RT-qPCR. The role of TMPO-AS1 in regulating miR-355-5p maturation in granulosa-like tumor (KGN) cells was analyzed by overexpression experiments. The interaction between TMPO-AS1 and premature miR-335-5p was analyzed by RNA pull-down assay. The subcellular location of TMPO-AS1 in KGN cells was analyzed by nuclear fractionation assay. The role of TMPO-AS1 and miR-335-5p in KGN cell proliferation was analyzed by BrdU assay.

**Results:**

TMPO-AS1 was increased in PCOS, while mature miR-355-5p was decreased in PCOS. TMPO-AS1 overexpression decreased mature miR-355-5p level but increased premature miR-355-5p. TMPO-AS1 was localized in both nucleus and cytoplasm. TMPO-AS1 directly interacted with premature miR-355-5p in KGN cells. TMPO-AS1 increased KGN cell proliferation while miR-355-5p decreased cell proliferation. The co-transfection assay showed that TMPO-AS1 reduced the suppressive effects of miR-355-5p on cell proliferation.

**Conclusions:**

TMPO-AS1 might suppress miR-335-5p maturation to participate in PCOS.

## Introduction

Polycystic ovary syndrome (PCOS) is a metabolic and endocrine disease due to hormonal disorders [1], which is mainly reflected by the increased androgen level. It affects premenopausal women [2], leading to prolonged or infrequent menstrual periods. Ovaries in PCOS patients may develop follicles and irregularly release eggs [3]. Without proper and timely treatment, POCS may lead to the development of acne scars, heart disease, and cancers [4, 5]. At present, treatments of PCOS mainly include lifestyle changes, healthy dietary structure, and medications [6, 7]. However, there is no cure for PCOS. Therefore, novel therapeutic approaches are needed to improve the treatment of PCOS.

Granulosa cells are the somatic cells of the sex cord and are closely associated with the development of eggs or oocytes [8]. Previous studies have clearly shown that the development of PCOS is at least partially caused by the altered proliferation and survival of granulosa cells [9–11]. Therefore, regulating granulosa cell proliferation is likely a potential therapeutic approach for PCOS [12, 13]. Although long non-coding RNAs (lncRNAs) and microRNAs (miRNAs) lack necessary genetic information for protein synthesis, they participate in human diseases by influencing protein synthesis [14], suggesting the role of lncRNAs as potential targets for PCOS treatment. In fact, certain lncRNAs and miRNAs have been proven to be critical regulators of granulosa cell behaviors and functions [12, 13]. Therefore, targeting the expression of lncRNAs and miRNAs may provide novel opportunities to develop anti-PCOS approaches. Unfortunately, the functionality of most lncRNAs and miRNAs in PCOS is still unknown.

TMPO-AS1 is a recently characterized oncogenic lncRNA in ovarian cancer, while its role in other ovary diseases is unknown [15]. In ovarian cancer, TMPO-AS1 increases LCN2 transcription by binding to transcription factor E2F6, thereby promoting cancer progression [15]. We performed a preliminary sequencing analysis and observed the altered expression of TMPO-AS1 in PCOS and its inverse correlation with miR-335-5p, which could negatively regulate granulosa cell proliferation [16]. Therefore, it is reasonable to hypothesize that TMPO-AS1 might interact with miR-335-5p to participate in PCOS. Therefore, we analyzed the interaction between TMPO-AS1 and miR-335-5p in PCOS.

## Materials and methods

### Participants and follicular fluid extraction

A total of 60 PCOS patients (20 to 32 years, 25.9 ± 3.1 years) who were admitted to Suzhou Wuzhong People’s Hospital between May 2019 and January 2021 were enrolled in the study after the approval was obtained from this hospital’s Ethics Committee. PCOS was diagnosed based on many approaches, including blood tests, symptoms, physical examinations, and pelvic ultrasound. It has been well established that altered protein and RNA levels in the follicular fluid surrounding the ovum may reflect the molecular defects of folliculogenesis in women with PCOS [1–3]. Therefore, vaginal ultrasound was performed to guide double lumen needles to extract follicular fluid. Follicular fluid was extracted from 60 controls (20 to 32 years, 25.8 ± 3.2 years) with suspected ovary disorders, which were excluded after further analysis. This study excluded patients with other complications. Informed consent was signed by all participants.

### Granulosa-like tumor cells and primary granulosa cells

A granulosa-like tumor (KGN) cell line COV434 was from Sigma-Aldrich and cultured in DMEM supplemented with 2 mM glutamine and 10% FBS at 37 °C in a humidified incubator with 5% CO_2_. Primary granulosa cells from one PCOS patient were isolated and cultured for cell proliferation assay.

### Cell transfections

COV434 cells were transfected with pcDNA3.1-TMPO-AS1 expression vector (Invitrogen) or miR-335-5p mimic (Sigma-Aldrich) using Neon Electroporation Transfection system (Thermo Fisher Scientific). The dosages of vector and miRNA were 10 μg and 40 nM for 10^6^ cells, respectively. Overexpression was confirmed every 24 h until 72 h.

### RNA preparation

Total RNA isolation from COV434 cells and follicular fluid samples was performed using Direct-zol RNA Kit (ZYMO RESEARCH). DNA removal from all RNA samples was performed using DNase I. All RNA samples were subjected to integrity analysis on 5% urea PAGE gel.

### RT-qPCRs

All RNA samples were reverse transcribed (RT) into cDNA samples and used for qPCRs with 18S rRNA as the internal control to determine the expression of TMPO-AS1 and premature miR-335-5p. Mature miR-335-5p expression was analyzed using All-in-One™ miRNA qRT-PCR Reagent Kits (Genecopoeia) with U6 as the internal control. Ct values were processed using the method of 2^−ΔΔCt^.

### Nuclear fractionation assay

Nuclear and cytoplasm of COV434 cells were prepared using the Nuclear/Cytosol Fractionation Kit (BioVision, #K266). After that, RNA isolation and RTs were performed, followed by semi-quantitative PCR with GAPDH as the internal control to determine TMPO-AS1 expression. PCR products were subjected to 1.5% agarose gel electrophoresis, and images were taken under UV lights after EB staining.

### RNA pull-down assay

Biotin ligated premature miR-335-5p (bio-miR-335-5p) and NC miRNA (bio-NC) were prepared by Invitrogen (Shanghai, China) and transfected into COV434 cells. Cell lysates were prepared 48 h later, followed by RNA pull-down using streptavidin agarose magnetic beads (Life Technologie). Following RNA isolation, RT-qPCRs were performed to determine TMPO-AS1 expression in these two pull-down samples.

### BrdU assay

Cell proliferation was analyzed and reflected by 5-Bromo-2-deoxyUridine (BrdU) incorporation. In brief, after transfection, both primary granulosa cells and COV434 cells were cultured for 72 h, followed by incubation with 10 mM BrdU (BD Pharmingen) in culture media for 2 h. Cells were fixed 30 min and incubated with peroxidase-coupled anti-BrdU-antibody (Sigma-Aldrich) for 60 min. The signals were visualized by incubation with peroxidase substrate for 30 min and determined by measuring OD values at 450 nm.

### Statistical analysis

Unpaired t test and ANOVA Tukey’s test were performed to compare two and multiple independent groups, respectively. Correlation analysis was performed with Pearson’s correlation coefficient. A *p* < 0.05 was deemed statistically significant.

## Results

### TMPO-AS1 and miR-355-5p expression and their correlation

**Fig. 1:**
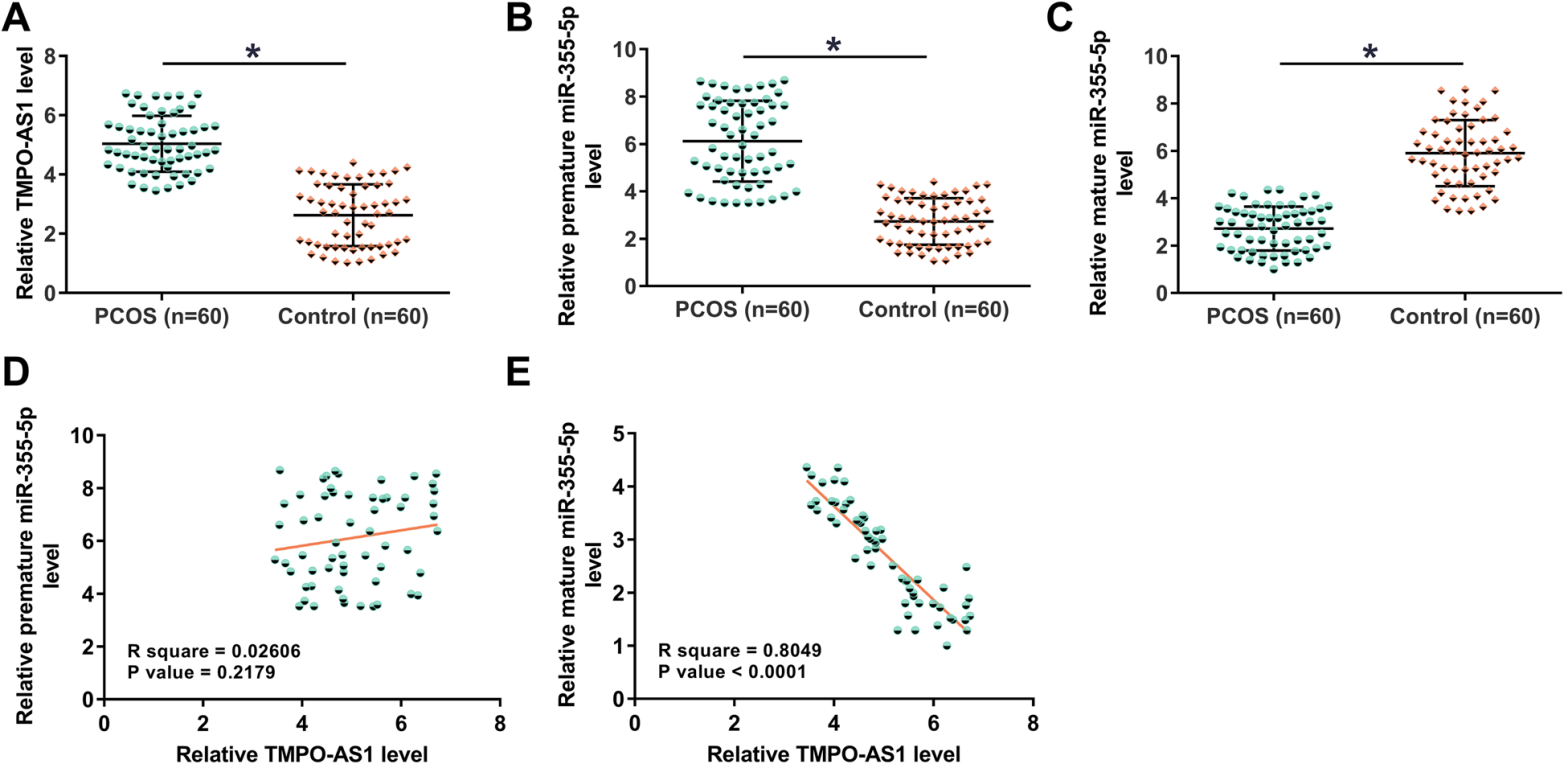
TMPO-AS1 and miR-355-5p expression and their correlation. Expression of TMPO-AS1 (**A**), premature miR-355-5p (**B**), and mature miR-355-5p (**C**) in follicular fluid samples from both PCOS and control groups was studied by RT-qPCR analysis. Correlations between TMPO-AS1 and premature miR-355-5p (**D**) and between TMPO-AS1 and mature miR-355-5p (**E**) were analyzed by Pearson’s correlation coefficient. *, *p* < 0.05

Expression of TMPO-AS1 and miR-355-5p (both mature and premature) in follicular fluid samples from both PCOS and control groups were measured using RT-qPCR. The results illustrated that TMPO-AS1 (Fig. 1A) and premature miR-355-5p (Fig. 1B) were increased in PCOS group (*p* < 0.05). In contrast, mature miR-355-5p was decreased in PCOS (Fig. 1C, *p* < 0.05). Therefore, TMPO-AS1 upregulation and inhibited miR-355-5p maturation may participate in PCOS. Correlation analysis illustrated that TMPO-AS1 was positively correlated with premature miR-355-5p, but the correlation was not statistically significant (Fig. 1D). In contrast, it was inversely correlated with mature miR-355-5p (Fig. 1E). Therefore, TMPO-AS1 is likely involved in miR-355-5p maturation.

### The subcellular location of TMPO-AS1 and its direct interaction with premature miR-355-5p

**Fig. 2:**
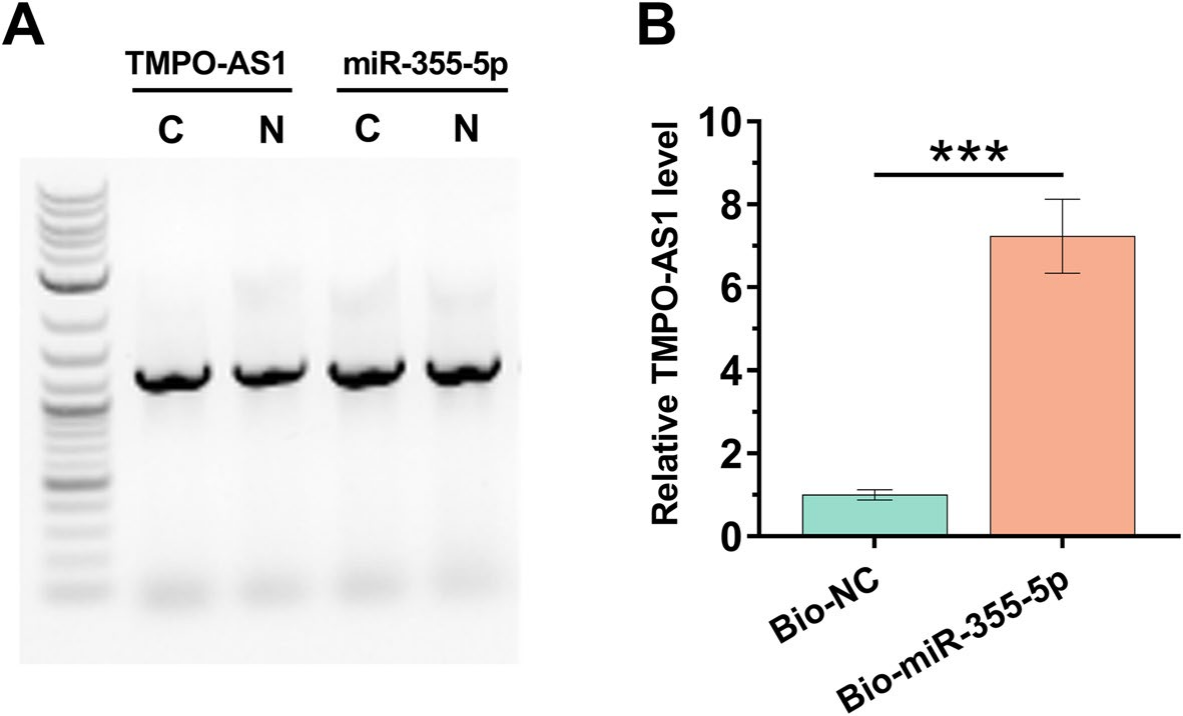
The subcellular location of TMPO-AS1 and its direct interaction with premature miR-355-5p. Nuclear fractionation assay was performed to analyze the subcellular location of TMPO-AS1 in COV434 cells (**A**). RNA pull-down assay was performed to analyze the direct interaction between TMPO-AS1 and premature miR-355-5p (**B**). N, nucleus; C, cytoplasm; *, *p* < 0.001

The subcellular location of TMPO-AS1 in COV434 cells was analyzed using nuclear fractionation assay. The data showed that TMPO-AS1 was expressed in both nuclear and cytoplasm samples (Fig. 2A). RNA pull-down assay was performed to analyze the direct interaction between TMPO-AS1 and premature miR-355-5p. Compared to bio-NC pull-down sample, TMPO-AS1 was significantly higher in bio-miR-355-5p pull-down sample (Fig. 2B, *p* < 0.001), suggesting a direct interaction between TMPO-AS1 and premature miR-355-5p. Therefore, TMPO-AS1 may interact with premature miR-355-5p in the nucleus, the primary location of premature miRNAs.

### The role of TMPO-AS1 in miR-355-5p maturation in COV434 cells

**Fig. 3:**
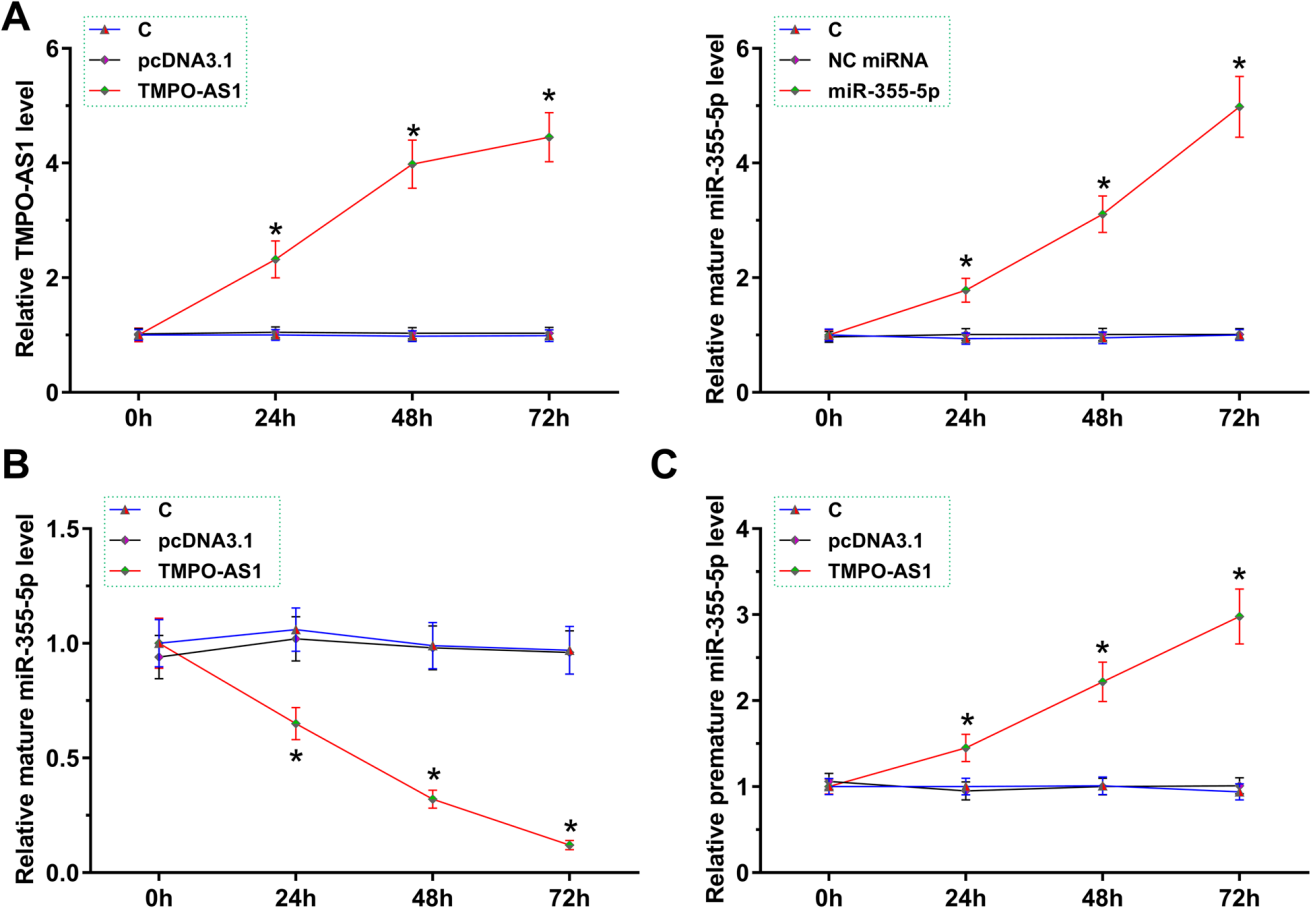
The role of TMPO-AS1 in miR-355-5p maturation in COV434 cells. COV434 cells were overexpressed with TMPO-AS1 or miR-355-5p, and the overexpression was confirmed every 24 h until 72 h (**A**). The effects of TMPO-AS1 overexpression on the expression of mature miR-355-5p (**B**) and premature miR-355-5p (**C**) was analyzed by RT-qPCR. *, *p* < 0.05

COV434 cells were overexpressed with TMPO-AS1 or miR-355-5p, and the overexpression was confirmed every 24 h until 72 h (Fig. 3A, *p*  < 0.05). Overexpression experiments followed by RT-qPCR analysis revealed that TMPO-AS1 overexpression decreased mature miR-355-5p level (Fig. 3B , *p* < 0.05) but increased premature miR-355-5p (Fig. 3C, *p* < 0.05). Therefore, TMPO-AS1 may suppress miR-355-5p maturation in COV434 cells.

### TMPO-AS1 increased both COV434 and primary granulosa cell proliferation through miR-355-5p

**Fig. 4:**
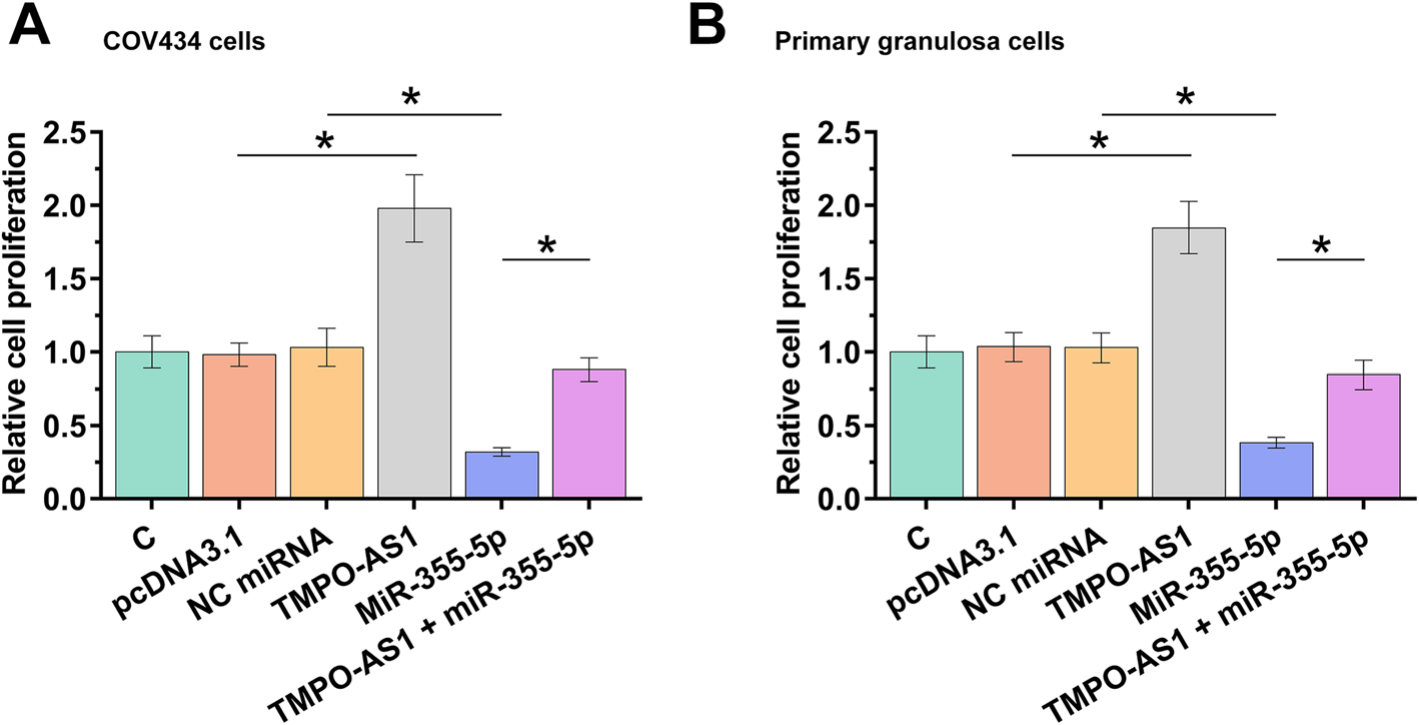
TMPO-AS1 increased COV434 and primary granulosa cell proliferation through miR-355-5p. BrdU assay was performed to analyze the role of TMPO-AS1 and miR-355-5p in the proliferation of both COV434 (**A**) and primary granulosa (**B**) cells. Cell proliferation was represented by OD values at 450 nm. *, *p* < 0.05

BrdU assay was performed to analyze the role of TMPO-AS1 and miR-355-5p in the proliferation of both COV434 (Fig. 4A) and primary granulosa (Fig. 4B) cells. TMPO-AS1 increased both COV434 and primary granulosa cell proliferation, while miR-355-5p decreased their proliferation. Co-transfection assay showed that TMPO-AS1 reduced the suppressive effect of miR-355-5p on cell proliferation (Fig. 4, *p* < 0.05).

## Discussion

The role of TMPO-AS1 in PCOS was explored in this study. We showed that TMPO-AS1 was highly expressed in PCOS. Functional analysis revealed that TMPO-AS1 suppressed miR-355-5p maturation to increase granulosa cell proliferation.

LncRNA TMPO-AS1 plays an oncogenic role in different cancers, especially in females [15, 16]. For instance, TMPO-AS1 is overexpressed in ovarian cancer and promotes cancer progression via increasing LCN2 transcription [15]. In addition, TMPO-AS1 is also overexpressed in cervical cancer and sponges miR-577 to increase RAB14 expression to enhance cancer progression. Based on our best knowledge, the involvement of TMPO-AS1 in other human diseases remains unclear. In this study, we showed that TMPO-AS1 was upregulated in PCOS. In addition, TMPO-AS1 overexpression increased granulosa cell proliferation. Therefore, TMPO-AS1 might promote granulosa cell proliferation in PCOS to affect disease progression. However, the exact role of TMPO-AS1 in the complicated pathogenesis of PCOS and its clinical applications remain to be further explored.

A recent study reported that miR-335-5p negatively regulated granulosa cell proliferation via SGK3 to affect PCOS [16]. Consistently, we showed miR-335-5p downregulation in PCOS and its inhibitory effects on cell proliferation. Interestingly, the premature miR-335-5p level was increased in PCOS. Therefore, the inhibited maturation of miR-335-5p, but not the suppressed transcription of miR-335-5p, participates in PCOS. Moreover, we found that TMPO-AS1 was closely correlated with mature miR-335-5p but not premature miR-335-5p. In addition, TMPO-AS1 overexpression decreased mature miR-335-5p expression but increased premature miR-335-5p expression [17]. Analysis of TMPO-AS1 subcellular location revealed that TMPO-AS1 is expressed in both the nucleus and the cytoplasm. It is known that premature miRNAs have to be transported from the nucleus to cytoplasm to form mature miRNA. Our data showed that TMPO-AS1 could directly interact with premature miR-335-5p. Therefore, TMPO-AS1 might absorb miR-335-5p in the nucleus to suppress its transportation, thereby inhibiting its maturation.

Our study characterized a novel TMPO-AS1/miR-335-5p axis in PCOS and showed it participates in PCOS by affecting granulosa cell proliferation, which plays a critical role in PCOS progression [9–11]. Therefore, regulating TMPO-AS1 and miR-335-5p expression may serve as a potential target to treat PCOS. However, animal model experiments and clinical trials are needed to confirm our conclusions. 

## Conclusion

TMPO-AS1 is overexpressed in PCOS and may suppress miR-335-5p maturation by directly interacting with premature miR-335-5p, thereby increasing cell proliferation to participate in PCOS.

## Data Availability

The data that support the findings of this study are not publicly available due to their containing information that could compromise the privacy of research participants but are available on request from the corresponding author.
